# Supplementary material for: Dual mechanism β-amino acid polymers promoting cell adhesion
Source: Nat Commun. 2021 Jan 25;12:562. doi: 10.1038/s41467-020-20858-x (PMC7835237; doi:10.1038/s41467-020-20858-x)
Supplement: Supplementary file 1 — Supplementary Information. [file 41467_2020_20858_MOESM1_ESM.pdf]

Supplementary Information

Dual mechanism β-amino acid polymers promoting cell adhesion

Qi Chen[1], Donghui Zhang[2], Wenjing Zhang[2], Haodong Zhang[2], Jingcheng Zou[2], Mingjiao Chen[3], Jin Li[3], Yuan Yuan[2], Runhui Liu[1,2]✉

[1]State Key Laboratory of Bioreactor Engineering, East China University of Science and Technology, Shanghai 200237, China.
[2]Key Laboratory for Ultrafine Materials of Ministry of Education, Frontiers Science Center for Materiobiology and Dynamic Chemistry, Research Center for Biomedical Materials of Ministry of Education, School of Materials Science and Engineering, East China University of Science and Technology, Shanghai 200237, China.
[3]Shanghai Key Laboratory of Orbital Diseases and Ocular Oncology, Department of Ophthalmology, Ninth People's Hospital, Shanghai Jiao Tong University School of Medicine, Shanghai 200011, China.
✉email: rliu@ecust.edu.cn

Materials

All chemical reagents and solvents were purchased from Adamas-beta® and used without further purification. Synthesized chemicals were purified using a SepaBean machine equipped with Sepaflash columns produced by Santai Technologies Inc. ^{1}H NMR and ^{13}C NMR spectra were collected on a 400 MHz or a 100 MHz Bruker spectrometer. UV/ozone cleaning machine (BZS250GF-TC) was from Shenzhen hwo technology Co., Ltd. The water used in these experiments was obtained from a Millipore water purification system with a minimum resistivity of 18.2 MΩ. cm. Maleimide-NH-OEG8-$CH_2CH_2COONHS$ ester was purchased from Biomatrik Inc. Multi-channel silicone coverslips containing 50 wells (103350) and 8 wells (103380) were purchased from Grace Biolabs (Bend, OR). RGDSPC and KRSRGYC peptides were obtained from Synpeptide in China. Fetal bovine serum (FBS) was obtained from Biological Industries. Alpha Minimum Essential Medium (α-MEM) and phosphate buffered saline (PBS) were purchased from HyClone (USA). MC3T3-E1 preosteoblast cells were obtained from the

Cell Bank of the Chinese Academy of Sciences (Shanghai, China). Mammalian cell LIVE/DEAD Viability/Cytotoxicity Kits (L3224) and alamarBlue Cell Viability Reagent were purchased from Thermo Fisher Scientific. Anti-fibronectin (ab2413), anti-laminin antibody (ab11575), and anti-collagen type I antibody (ab34710) were purchased from Abcam (Cambridge, MA). Human plasmin (Cat: 527621-10U), heparinase type I (batch: 20170707), type II (batch: 20170707), and type III (batch: 20170707) were purchased form Sigma-Aldrich. Collagenase type I (Cat: 17100-017) and type II (Cat: 17100-015) were purchased from Gibco. Anti-vitronectin antibodies (bs-1932R) and Alexa Fluor-555-conjugated anti-vinculin antibody (Lot: AE041534) was purchased from Beijing Biosynthesis Biotechnology Co., Ltd. FITC-phalloidin and 4'-6-diamidino-2-phenylindole (DAPI, Lot: 096M4014V) were purchased from Yeasen Biotech Co., Ltd. Recombinant human Integrin αvβ3 (Lot: OMM1518021) was purchased from R&D Systems, Inc. (R&D, USA). NanoOrgane Protein Quantitation Kit (N666) was purchased from Invitrogen. GelMA (EFL-GM-90) was purchased from Suzhou Intelligent Manufacturing Research Institute, China. PLA (Mat-No. 99104565) was purchased from Evonik Specialty Chemicals Co., Ltd.

Supplementary Table 1. Surface Element content measured by XPS.

	%C	%O	%N	C:N
Glass	16.85	55.08	4.26	-
NH_2-Glass	50.89	25.67	7.76	6.5
OEG8	38.95	38.89	4.71	8.2
$DM_{50}CO_{50}$	52.3	28.16	7.61	6.8

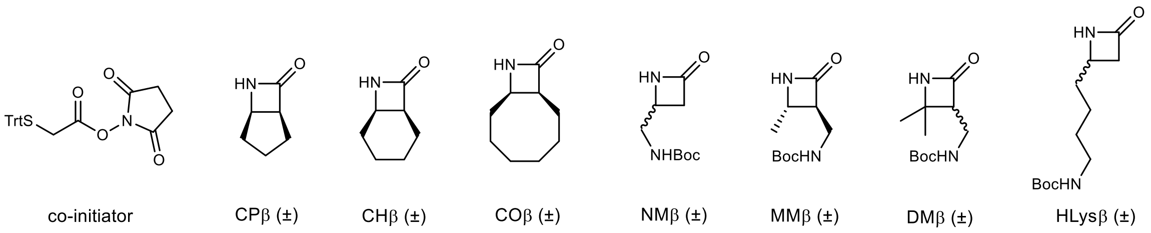

Supplementary Figure 1. Chemical structure of the co-initiator and β-lactam monomers.

The co-initiator and all β-lactam monomers (Scheme 1) were synthesized by following the previously reported methods[1-3]. All monomers are racemic mixtures.

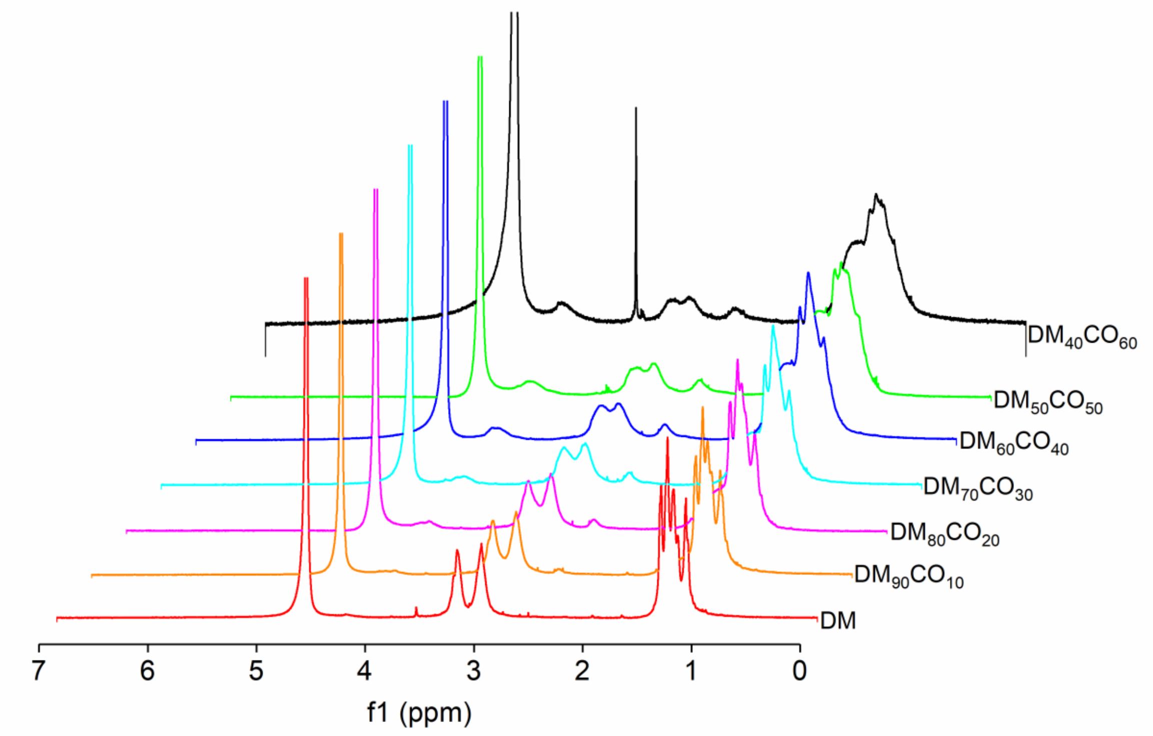

Supplementary Figure 2. ^{1}H NMR spectra of DM:CO series polymers with variable ratio of two subunits.

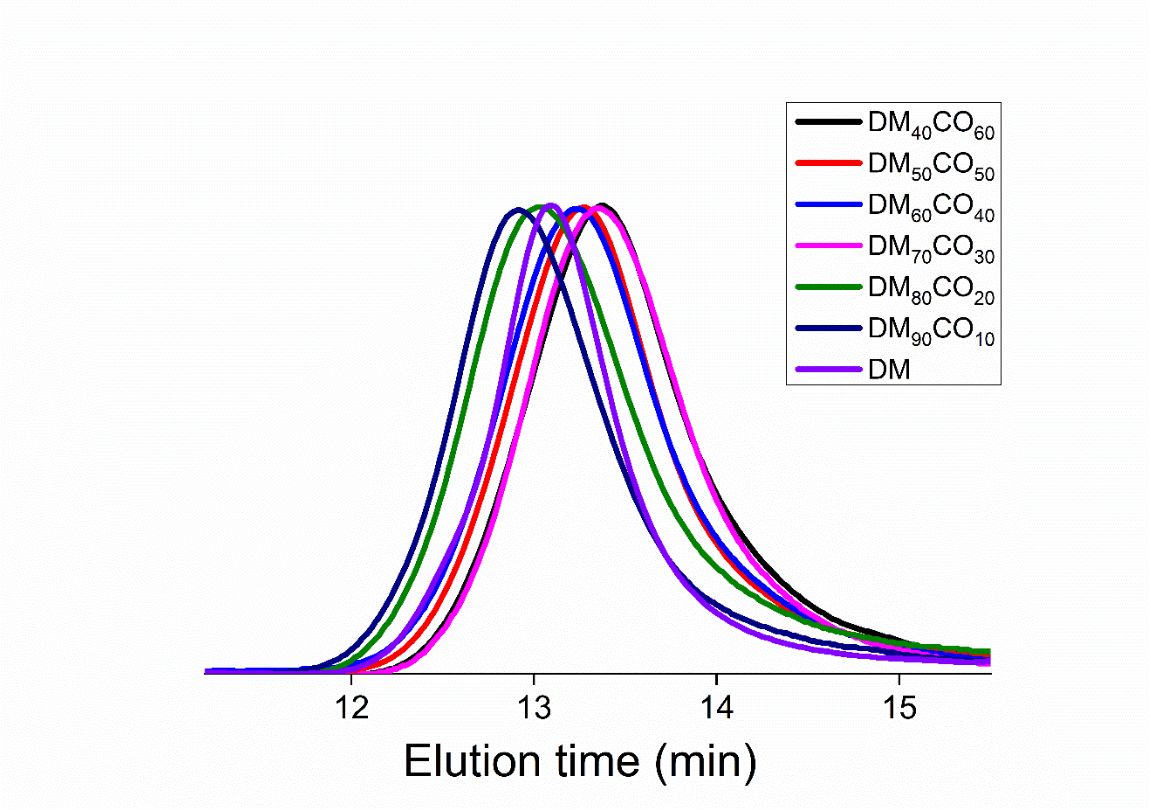

Supplementary Figure 3. GPC curves of DM:CO series polymers with variable ratio of two subunits.

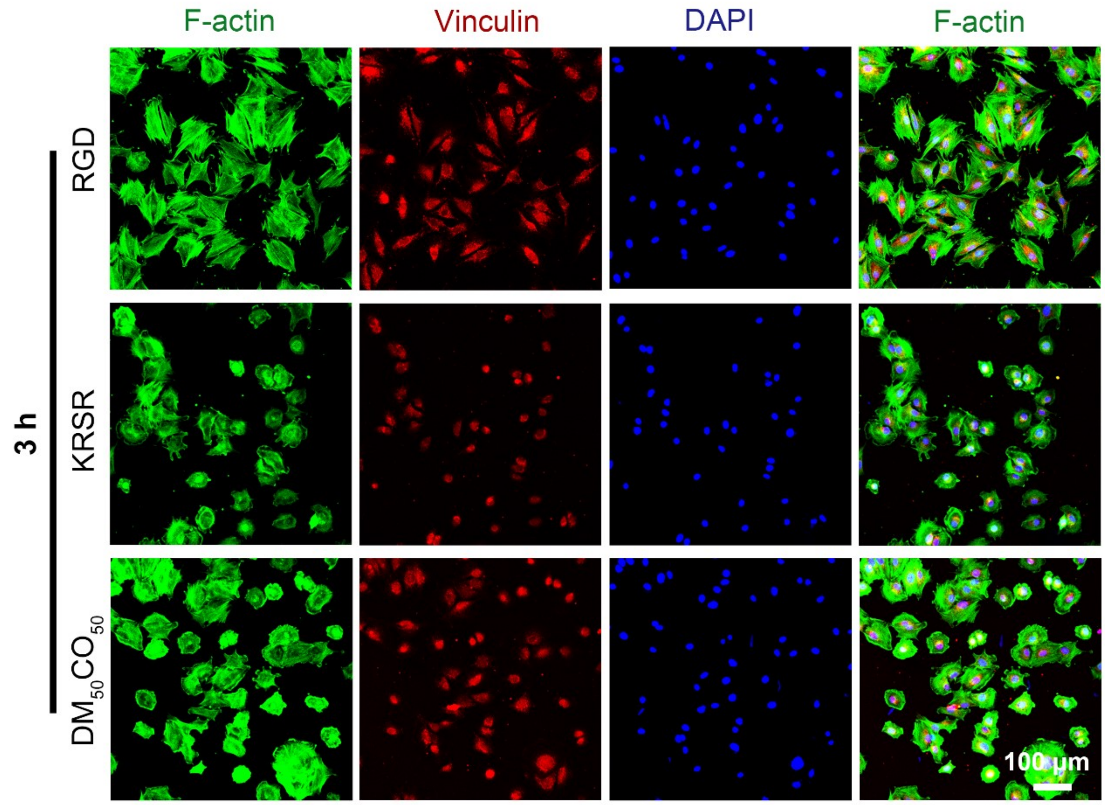

Supplementary Figure 4. Confocal images (green, actin; red, vinculin; blue, nucleus) of preosteoblast cells cultured on RGD, KRSR and $DM_{50}CO_{50}$-modified surfaces for 3 h. Scale bars: 100 μm.

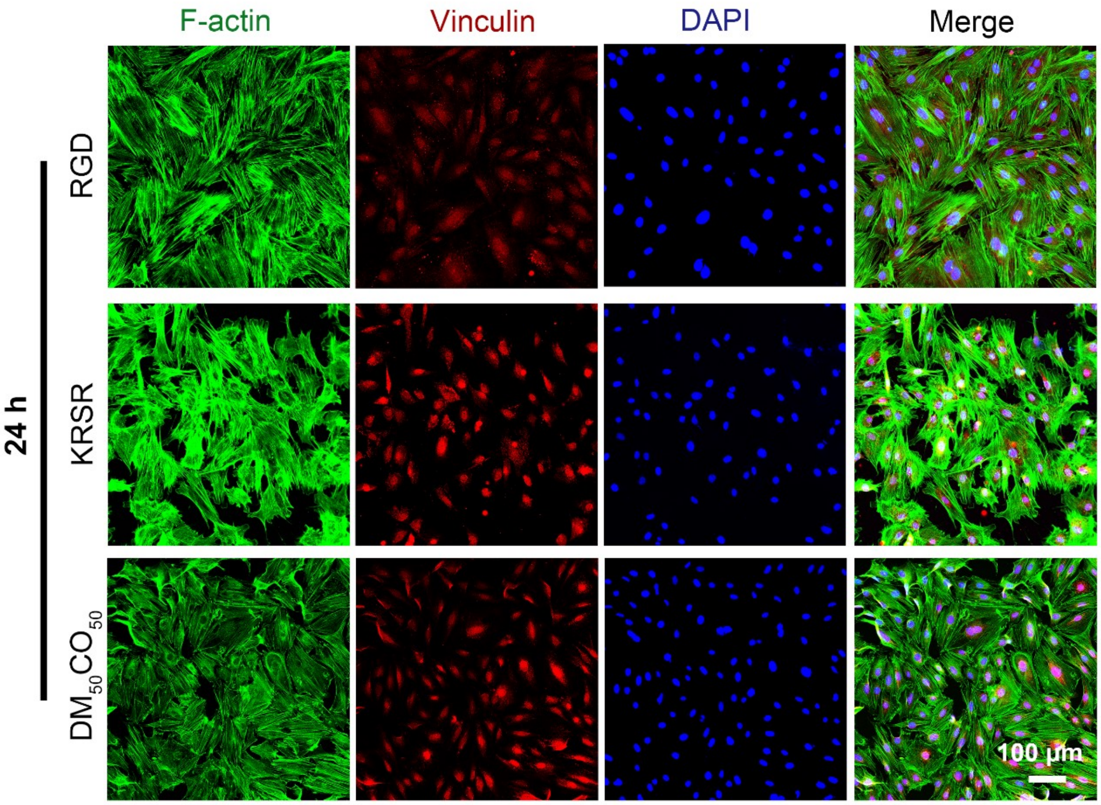

Supplementary Figure 5. Confocal images (green, actin; red, vinculin; blue, nucleus) of preosteoblast cells cultured on RGD, KRSR and $DM_{50}CO_{50}$-modified surfaces for 24 h. Scale bars: 100 µm.

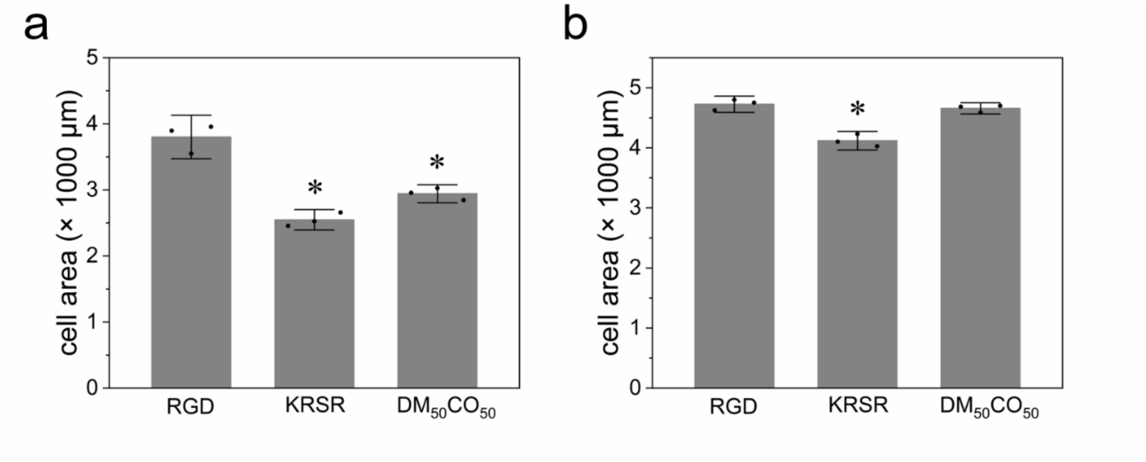

Supplementary Figure 6. Cell area of preosteoblast cells cultured on RGD, KRSR and $DM_{50}CO_{50}$-modified surfaces for 3h (a) and 24 h (b), respectively. Data represent the mean ± s.d. (n = 3). Statistical analysis: one-way ANOVA with Tukey post-test, *$p < 0.05$.

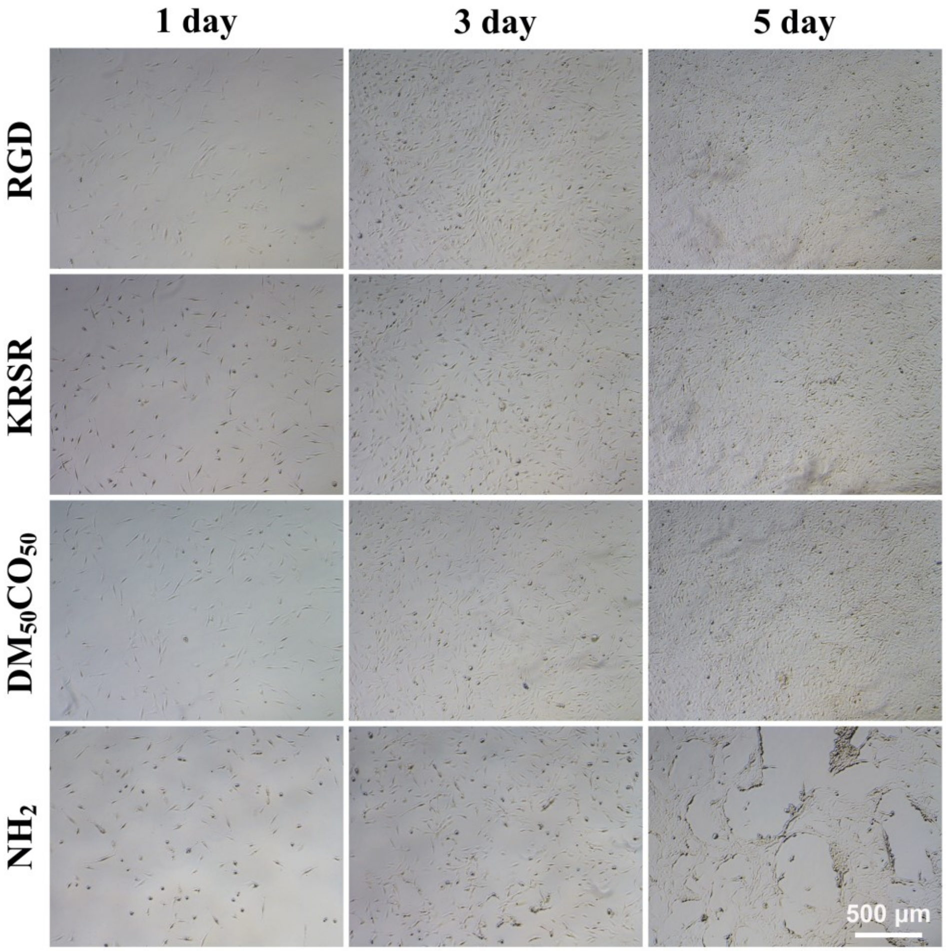

Supplementary Figure 7. Bright field images of preosteoblast cells cultured on NH2, RGD, KRSR and $DM_{50}CO_{50}$-modified surfaces for 1, 3, 5 days. Scale bar: 500 µm.

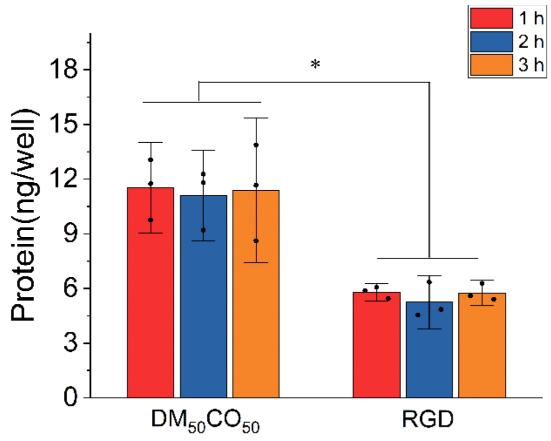

Supplementary Figure 8. Quantification of adsorbed serum protein on $DM_{50}CO_{50}$ and RGD-modified surfaces after incubation with serum-containing cell culture media for 1, 2 and 3 hours. Data represent the mean ± s.d. (n = 3). Statistical analysis: one-way ANOVA with Tukey post-test, *$p < 0.05$.

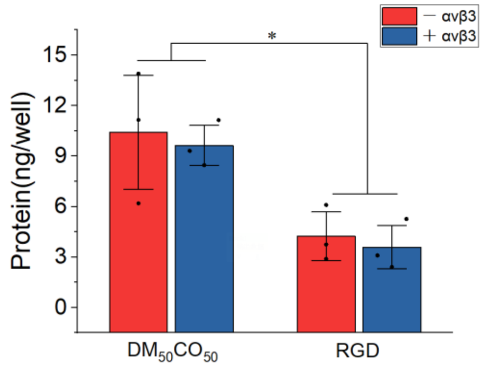

Supplementary Figure 9. Quantification of adsorbed serum protein on $DM_{50}CO_{50}$ and RGD-modified surfaces that were incubated with serum-containing media first and then treated with or without integrin αvβ3 before cell seeding. Data represent the mean ± s.d. (n = 3). Statistical analysis: one-way ANOVA with Tukey post-test, *$p < 0.05$.

Supplementary References

1. Liu, R.; Chen, X.; Falk, S. P.; Mowery, B. P.; Karlsson, A. J.; Weisblum, B.; Palecek, S. P.; Masters, K. S.; Gellman, S. H., Structure-activity relationships among antifungal nylon-3 polymers: identification of materials active against drug-resistant strains of Candida albicans. *J Am Chem Soc* **2014,** *136* (11), 4333-42.
2. Qian, Y.; Qi, F.; Chen, Q.; Zhang, Q.; Qiao, Z.; Zhang, S.; Wei, T.; Yu, Q.; Yu, S.; Mao, Z.; Gao, C.; Ding, Y.; Cheng, Y.; Jin, C.; Xie, H.; Liu, R., Surface Modified with a Host Defense Peptide-Mimicking beta-Peptide Polymer Kills Bacteria on Contact with High Efficacy. *ACS applied materials & interfaces* **2018,** *10* (18), 15395-15400.
3. Eldred, S. E.; Pancost, M. R.; Otte, K. M.; Rozema, D.; Stahl, S. S.; Gellman, S. H., Effects of side chain configuration and backbone spacing on the gene delivery properties of lysine-derived cationic polymers. *Bioconjugate chemistry* **2005,** *16* (3), 694-699.
